# Serotyping and Antimicrobial Resistance Profiling of Multidrug-Resistant Non-Typhoidal *Salmonella* from Farm Animals in Hunan, China

**DOI:** 10.3390/antibiotics12071178

**Published:** 2023-07-12

**Authors:** Zhuohui Zhang, Jiyun Li, Rushun Zhou, Qianqian Xu, Shiyin Qu, Hongguang Lin, Yan Wang, Pishun Li, Xiaofeng Zheng

**Affiliations:** 1 College of Veterinary Medicine, Hunan Agricultural University, Changsha 410128, China; zh_zhang@stu.hunau.edu.cn (Z.Z.); lijiyuny@foxmail.com (J.L.);; 2 Hunan Engineering Technology Research Center of Veterinary Drugs, Hunan Agricultural University, Changsha 410128, China; 3 Hunan Provincial Institution of Veterinary Drug and Feed Control, Changsha 410006, China

**Keywords:** chicken farm, foodborne pathogen, horizontal transmission, multidrug resistance, pig farm, *Salmonella typhimurium* monophasic variant, vertical transmission

## Abstract

Non-typhoidal *Salmonella* (NTS) is a foodborne pathogen and a prevalent causative agent for disease outbreaks globally. The *Salmonella* enterica serovar 4,[5],12:i:- (S.4,[5],12:i:-) belongs to the monophasic variant of *Salmonella typhimurium*, which is of current global concern. In this study, the epidemiology and genomic characterization of S. 4,[5],12:i:- isolates from 17 livestock farms in Hunan Province between 2019 and 2020, as well as their susceptibility to 14 antimicrobial agents, were profiled. Twelve *Salmonella* serotypes were identified using the White–Kauffmann–Le Minor scheme, and whole-genome sequencing analyses were conducted based on these isolates. Overall, 107 *Salmonella* strains were isolated, of which 73% (78/107) were multidrug resistant. Resistance to tetracycline (85.05%) was found to be the most prevalent, followed by the *oqxAB* and *aac(6′)-Ib-cr* genes. *S. typhimurium* (monophasic) 4,[5],12:i:- was the most common serotype, followed by *S. typhimurium* and *S. derby.* Most antimicrobial-resistant strains were isolated from pigs, indicating that they could be important reservoirs of resistant non-typhoidal *Salmonella* strains. The presence of similar genetic environments in S. 4,[5],12:i:- indicates both vertical and horizontal transmission of resistance plasmids, which may promote the spread of drug resistance genes. Appropriate measures should be taken to curb the prevalence of S. 4,[5],12:i:-.

## 1. Introduction

*Salmonella* is a Gram-negative, rod-shaped bacterium that is facultatively anaerobic and belongs to the Enterobacteriaceae family. These bacteria are generally mobile, without a capsule, non-spore-forming, and are able to colonize the digestive tracts of many vertebrates. *Salmonella* is one of the most important zoonotic pathogens and a causative agent for food-borne gastroenteritis in humans, domestic animals, and wildlife worldwide [1]. In the USA, *Salmonella* was estimated to cause illness in 46,623 patients annually across 53 states in 2016 [2]. Salmonellosis is the second most commonly reported gastrointestinal infection in the European Union (EU), with 91,662 confirmed human salmonellosis cases in all member states in 2017 [3]. *Salmonella* is also reportedly responsible for approximately 70–80% of foodborne pathogenic outbreaks in China [4].

*Salmonella* can be classified as typhoidal or non-typhoidal (NTS) based on its ability to cause specific pathologies in humans [5]. It is notable that non-typhoidal *Salmonella* is the main pathogen causing diarrhea and responsible for approximately 153 million cases of gastroenteritis as well as 57,000 deaths globally per annum [6]. Recently, the incidence of non-typhoidal salmonellosis was reported to be 626.5 cases per 100,000 persons in China [7].

Generally, animal farms are cultivable environments for the replication and persistence of *Salmonella*, and livestock are considered to be natural reservoirs for this bacterium [8]. Because fluoroquinolones, cephalosporins, azithromycin, and carbapenems are critically important antibiotics for the treatment of salmonellosis, emerging resistances to these drug classes are of paramount concern [9]. As a result, the World Health Organization (WHO) has deemed fluoroquinolone-resistant *Salmonella* to be priority pathogens, which urgently calls for new antimicrobials [10].

More than 2600 distinct serovars of *Salmonella* have been identified. In Salmonella, the resistance profiles vary in different serovars [11]. However, in recent decades, the monophasic variant of *S. typhimurium*, has emerged as a new multidrug-resistant serovar. This variant lacks the second-phase flagellar antigen (encoded by *fljB*) and produces a unique antigenic formula 4,[5],12:i:-. It has been frequently isolated from husbandry animals, food products, and humans in many countries and territories worldwide [12] and has become one of the major serotypes responsible for human diarrhea. In 2017, *S. ntyphimurium* (monophasic) was the third most commonly identified serovar among human cases of salmonellosis reported in the European Union after *S. enteritidis* and *S. typhimurium* [3]. A recent study pointed out that the prevalence of S. 4,[5],12:i:- has increased and become the second most frequently identified serotype in outpatients in the Henan Province of China [13]. 

To provide a further understanding of multidrug resistance and the distribution of drug resistance genes in *S. typhimurium* (monophasic), we collected 107 *Salmonella* isolates from 17 livestock farms (9 chicken farms and 8 pig farms) in Hunan Province, China. Serological typing and molecular epidemiological analyses were used to comprehensively profile the diversity of *Salmonella* isolates in their serotypes, drug resistances, and genotypic characteristics. These data will aid in the development of scientific strategies for the future prevention and control of *Salmonella*.

## 2. Results

### 2.1. Salmonella Strains Isolation

A total of 107 non-typhoidal *Salmonella* isolates were collected, with an isolation rate of 5.1% (52/1017) in samples from chickens and 4.5% (55/1223) in samples from pigs, in 2019 and 2020; the isolation rate in 2019 (7.8%) was higher than that in 2020 (2.8%) (Table 1). The 107 collected isolates were sampled in geographically different sites, as follows: Changsha (*n* = 15); Liuyang (*n* = 20); Ningxiang (*n* = 22); Changde (*n* = 30); Zhuzhou (*n* = 10); Chenzhou (*n* = 6); Leiyang (*n* = 3); and Xiangtan (*n* = 1) (Supplementary Materials).

### 2.2. Prevalence of Salmonella Serovar

The 12 serovars were identified in 107 isolates (Table 2), including *S. typhimurium* (monophasic) (*n* = 34), *S. typhimurium* (*n* = 21), and S. derby (*n* = 17). Others serovars included *S. rissen* (*n* = 13), *S. enteritidis* (*n* = 11), *S. kentucky* (*n* = 2), *S. Iindiana* (*n* = 2), *S. london* (*n* = 1), *S. thompson* (*n* = 1), *S. meleagridis* (*n* = 1), and *S. readings* (*n* = 1). The dominant serotypes in chickens were *S. typhimurium* (28.9%), and *S. enteritidis* (21.2%), while the dominant serotypes in pigs were *S. typhimurium* (monophasic) (49.1%) and *S. derby* (21.8%) (Supplementary Materials).

### 2.3. Antibiotic Susceptibility Testing

Fourteen antibiotics were selected for the susceptibility test, including tetracycline, ampicillin, amoxicillin/clavulanic acid, ceftazidime, ceftiofur, gentamicin, florfenicol, enrofloxacin, ofloxacin, spectinomycin, gentamicin, meropenem, trimethoprim/sulfamethoxazole, sulfafurazole, and colistin (Table 3). The results showed that 73% (78/107) of the *Salmonella* isolates were resistant to three or more antimicrobial agents. The *Salmonella* strains were mostly found to be resistant to tetracycline (84.1%), followed by ampicillin (70.9%), florfenicol (66.4%), sulfafurazole (63.6%), spectinomycin (62.6%), trimethoprim/sulfamethoxazole (56.1%), enrofloxacin (43.9%), gentamicin (34.6%), ofloxacin (15.9%), and colistin (10.3%). However, all isolates were less frequently resistant to ceftiofur (8.4%), ceftazidime (5.6%), amoxicillin (4.7%), and meropenem (1.9%) (Table 2). Interestingly, resistance to ceftazidime (*n* = 2), meropenem (*n* = 2), and colistin (*n* = 11) was observed only in *Salmonella* isolated from chickens. Overall, the multidrug-resistant profile of the pig-origin *Salmonella* (*n* = 51) was higher than that of chicken-origin *Salmonella* (*n* = 27). In addition, the resistance rates of the pig-derived strains to six drugs (tetracycline, florfenicol, sulfafurazole, trimethoprim/sulfamethoxazole, enrofloxacin, and colistin) were all higher when compared with the chicken-derived strains (Table 4).

### 2.4. Antibiotic Resistance Gene and Plasmid Profiles

**Figure 1:**
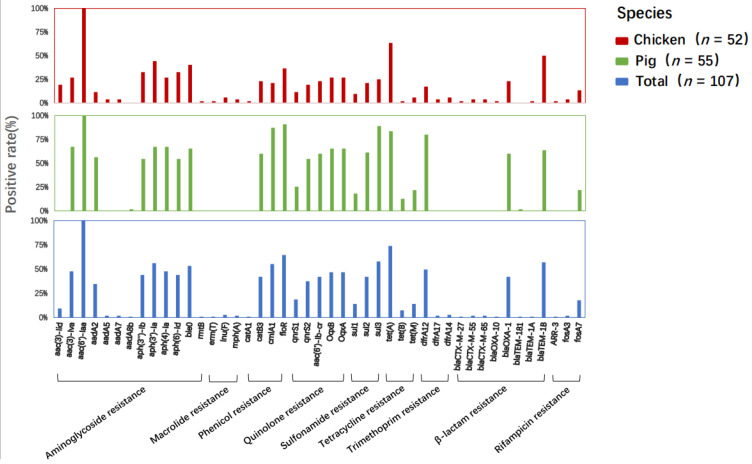
Different positive rates of resistance genes between chickens and pigs.

**Figure 2:**
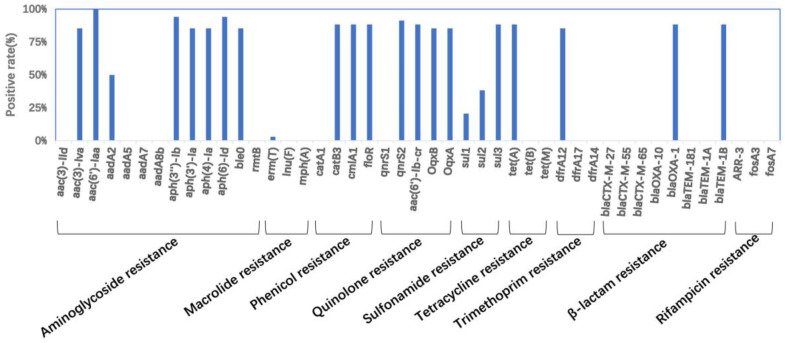
Positive rates of diverse resistance genes among different S. 4,[5],12:i:- isolates.

A total of 46 antimicrobial resistance genes (ARGs) were detected in the *Salmonella* isolates (Figure 1). Most ARGs (e.g., *aac(6′)-Iaa*, *oqxAB*, *aac(6′)-Ib-cr*, *qnrS1*, and *qnrS2*) are associated with resistance to aminoglycosides and quinolones. The aminoglycoside gene *aac(6′)-Iaa* was detected in all isolates. In addition, three plasmid-mediated quinolone resistance (PMQR) genes [*oqxA* (*n* = 50), *oqxB* (*n* = 50), *aac(6′)Ib-cr* (*n* = 45)] and two variants of the qnrS gene family *qnrS2* (*n* = 40) and *qnrS1* (*n* = 20) were detected. Among the genes encoding β-lactamases, the majority of CTX-M-type genes were *blaCTX-M-27*, *blaCTX-M-55* and *blaCTX-M-65*. The frequency of occurrence of the majority of CTX-M-type genes was lower than 4.7%, which is consistent with the finding of less resistance to β-lactam antibiotics in all isolates (Figure 1 and Figure 2). The *Salmonella* isolates that were positive for the tetracycline resistance gene *tet(A)* accounted for 73.8% and carried the sulfonamide resistance gene *sul2* (42%) (Figure 2). Additionally, the sulfonamide resistance gene sul3 was detected in more than half of the isolates.

The results of the plasmid replicons in 107 *Salmonella* isolates are presented in the Supplementary Materials. The results show that the most abundant plasmid replicons were IncHI2 (33.6%, 36/107), IncHI2A (33.6%, 36/107), IncX1 (15.9%, 17/107), IncFII (S) (15.0%;16/107), and IncFIB(S) (15.0%, 16/107). 

### 2.5. Characteristic of S. 4,[5],12:i:-

Among all isolates, 34 S. 4,[5],12:i:- isolates (31.8%, 34/107) were identified according to serotyping. Isolates with similar sequence types were grouped based on their phylogenetic relationship, and all S. 4,[5],12:i:- isolates belonged exclusively to ST34 (Supplementary Materials). It was the most common serovar in our sampling (Table 4), particularly from pigs. An antimicrobial susceptibility test of these 34 S. 4,[5],12:i:- isolates against 14 antimicrobial compounds showed resistance phenotypes in all isolates. The most common resistance was to tetracycline (100%), followed by spectinomycin (91.2%), florfenicol (91.2%), ampicillin (91.2%), trimethoprim/sulfamethoxazole (88.2%), enrofloxacin (76.5%), sulfafurazole (70.6%), gentamicin (47.1%), and ofloxacin (20.6%). The S. 4,[5],12:i:- isolates were less frequently resistant to ceftiofur (2.9%) and colistin (2.9%), and none of them were resistant to amoxicillin/clavulanic acid, meropenem, or ceftazidime (Table 5).

### 2.6. Phylogenetic Analysis

**Figure 3:**
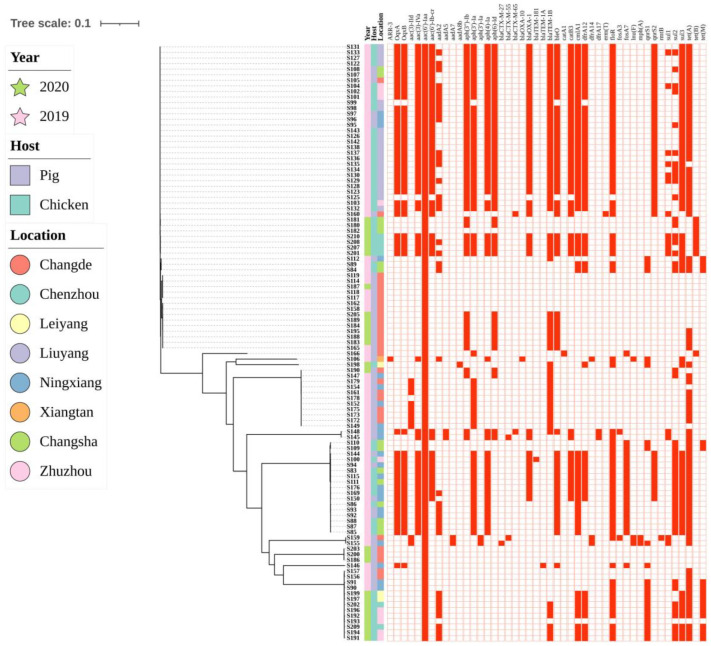
Bayesian phylogenetic analysis of 107 isolates (**left**) and the correlation with the distribution of resistance genes (**right**). Location and time are represented in the middle. To further evaluate possible transmission events, we coded the farms in the same city with one color (defined as intra-farm association events).

To investigate the genomic relationships among the isolates, a phylogenetic tree was established based on core single-nucleotide polymorphism (SNP) analysis (Figure 3). Although there were differences in the hosts, timing, and sites of the collection, these strains still exhibited relatively close genetic relationships. The close genetic relationship between the isolates at different time points on the same farm or city also proved the existence of clonal transmission. For example, the high similarity in genomic data in strains S90, S91, S156, S157, and S191–S193 conceivably indicated that certain hosts had driven the horizontal transmission. It is concerning that clonal transmission probably happened alongside horizontal transmission in this study.

## 3. Discussion

Poultry and livestock farms are considered to be favorable biotopes for the accumulation of pathogens like *Salmonella*, which cause huge economic losses in many countries, including China [14]. Hunan Province is one of the most concentrated areas for farm animals in China [15]. Disease control and prevention during breeding mainly depend on the use of antibiotics. The irrational use of antibiotics, however, has contributed to the emergence of multidrug-resistant bacteria under selective antimicrobial pressure. 

In this study, resistance phenotypes varied in the identified isolates, as 73% were resistant to at least three classes of antimicrobials (considered multidrug-resistant), which is remarkably higher than that observed in previous reports conducted in China [7] and lower than the resistance rates reported in Argentina and Australia [16,17]. Most of the resistant strains were isolated from pigs, which indicates that pigs could be important reservoirs of resistant non-typhoidal *Salmonella* strains. Among the identified drug resistances, resistance to tetracycline (85.1%) and ampicillin (70.1%) was predominant. These results are in agreement with previous studies on *Salmonella* isolates obtained from food animal farms in Xinjiang, China [18]. Resistance to quinolones and β-lactams was also recognized in many *Salmonella* isolates in this study, which is in agreement with previous reports [19,20]. Quinolone resistance, and that to ciprofloxacin in particular, has become a common issue in China and other countries, particularly resistance to ciprofloxacin [21]. The high prevalence of such genes is regarded as a significant threat to public health since these antimicrobials are currently used for frontline therapy against salmonellosis in humans [22].

PMQR genes are very common in farms [23], and this study showed that *oqxAB* and *aac(6′)-Ib-cr* were the main PMQR genes. All *oqxA*-positive isolates were screened for *oqxB*. *oqxAB*, and *aac(6′)-Ib-cr* commonly coexisted in the same strain, and 36 strains were found to carry them simultaneously. Carrying two or more PMQR genes in the same strain normally leads to resistance to nalidixic acid and decreases the susceptibility to fluoroquinolones like ciprofloxacin [24]. The presence of different ARGs based on whole-genome sequencing analysis demonstrated that the *aac(6′)-Iaa* gene, which mediates the resistance to aminoglycosides, was detected in all of the studied isolates. This was consistent with previous Chinese and South Korean studies [25]. The high levels of resistance to quinolones detected in this study may be due to the acquisition of PMQR genes through horizontally transferable elements, as well as mutagenesis in genes affecting the DNA gyrase and DNA topoisomerase IV genes [26].

Determination of serovars and multilocus sequence typing (MLST) patterns showed the dominance of S. 4,[5],12:i:- among the collected *Salmonella* isolates, especially those from pigs. This is consistent with the results of previous studies, in most of which S. 4,[5],12:i:- strains originated from pigs and pork products [27]. Intriguingly, the isolates from other sources such as chicken or the environment were less resistant to antibiotics of clinical importance, suggesting that pigs might be important reservoirs of resistant S.4,[5],12:i:- strains. The multidrug resistance of S. 4,[5],12:i:- is primarily associated with antimicrobials from seven classes. The detection of the *aac(6′)-Ib-cr*, *oqxA*, *oqxB*, *qnrS1*, and *qnrS2* genes has been reported in different serovars, including isolates of S. 4,[5],12:i:-, S. London, S. Indiana, S. Thompson, S. Kentucky, and S. Enteritidis. The presence of these genes enhances the adaptability of S. 4,[5],12:i:- to drugs, promoting the broader dissemination of such resistance genes [23]. In this study, 13 different plasmid replicons were identified among 107 *Salmonella* isolates. The most abundant plasmids were IncHI2A, IncHI2, and IncX1 (Supplementary Materials). IncHI2A and IncHI2 were predominant in S.4,[5],12:i:-. Interestingly, these plasmids were found to be associated with resistance to different antimicrobial classes, including β-lactams, aminoglycosides, sulfafurazole, tetracyclines, and polymyxins [28]. Consequently, these plasmids may increase the risk related to the horizontal transmission of these antimicrobial resistance genes in animal food chains, leading to severe disease in humans [29].

Phenotypic and genotypic resistance of most tested antibiotics showed high coherence, but tetracycline and quinolone resistance showed moderate coherence. The obtained results are consistent with previous reports on *Salmonella* isolates from dead poultry that revealed that drug resistance gene expression patterns and drug resistance spectra were remarkably similar among strains in Shandong [14]. Similarly, a large-scale study reported high levels of coherence between phenotypic and genotypic resistance for all tested antibiotics [30]. Hence, performing phenotypic verification on the collected isolates was necessary to avoid potential bias caused by genomic analysis. 

Phylogenetic analysis showed that isolates of the same serovar with similar sequence types were closely clustered. Notably, the exact inter-farm transmission event occurred among ST34, which may be due to farms’ lack of strict hygiene standards for handling. Hence, it is essential to improve hygiene and sanitizing procedures. Additional documentation of the traceability of inputs and outputs that may carry disease sources on each farm can help reduce the persistence and spread of *Salmonella* between poultry farms. However, we found that the detection rates of isolates and drug resistances were lower in 2020 than in 2019; one potential explanation is that the Ministry of Agriculture of the People’s Republic of China formulated plans to ban or reduce the use of specific antimicrobials in 2019 [7]. This indicates that strengthening veterinary medicine management could help to effectively prevent the development of antibiotic-resistant bacteria. 

In conclusion, in this study, the high prevalence of multidrug-resistant non-typhoidal *Salmonella* in the studied samples and its severe risk to human health were reported. The results indicate that, in the future, we must continue monitoring *Salmonella* serovars and conduct strategic control plans based on whole-genome sequencing. The application of an antimicrobial management plan for the rational use of essential antimicrobials in animal farms will also be vital to help control the spread and prevalence of drug resistance genes and to provide reliable human health protection measures.

## 4. Materials and Methods

### 4.1. Sample Collection and Isolation of Salmonella Strains

In 2019 and 2020, 2240 fecal samples were collected from 17 chicken and pig farms located in nine cities of Hunan Province: Changsha, Leiyang, Hengyang, Xiangtan, Ningxiang, Changde, Zhuzhou, Chenzhou, and Liuyang. *Salmonella* was isolated as described previously [19,20]. Briefly, cotton swab samples were subjected to pre-enrichment in buffered peptone water and then enriched in a modified semisolid Rappaport Vassiliadis plate, and colonies were isolated on xylose lysine deoxy-cholate agar. Subsequently, the isolated strains were confirmed via the amplification of the invA gene using the following primers according to a previously described protocol [31]: F:5′-ACAGTGCTCGTTTACGACCTGAAT-3′ and R:5′-AGACGACTGGTACT-GATCGATAAT-3′. Specifically, 25 PCR cycles with an annealing temperature of 56 °C were performed with Taq Polymerase (Tsingke Biotechnology Co., Ltd., Beijing, China) on a thermal cycler. Colonies confirmed as *Salmonella* were inoculated into Luria–Bertani broth for minimal inhibitory concentration (MIC) determination and genomic DNA preparation.

### 4.2. Detection of the Salmonella Strain Serotypes

The confirmed isolates were serotyped via slide agglutination using commercially available antisera kits (Tianrun BioPharmaceutical Co. Ltd., Ningbo, China) with O and H antigen-specific sera. Serovar results were interpreted according to the Kauffmann–White–Le Minor scheme [32].

### 4.3. Antibiotic Susceptibility Testing

The MIC of 14 antibiotics (nine classes) was determined using the microdilution broth method according to the criteria recommended by the Clinical and Laboratory Standards Institute [33]. *Escherichia coli* ATCC25922 was used as a quality control strain. 

### 4.4. Whole-Genome Sequencing, De Novo Assembly, and Annotation

Genomic DNA was extracted from the *Salmonella* isolates and purified using the TIANamp Bacteria DNA Kit (Tiangen Biotech Co., Beijing, China) according to the manufacturer’s instructions. Protein quality was assessed via gel electrophoresis and quantified using a Qubit Fluorometer 2.0 (Invitrogen, Waltham, MA, USA; Life Technologies, Carlsbad, CA, USA). Whole genome sequencing was performed using Annoroad Gene Technology (Beijing, China) on a NovaSeq 6000 S4 sequencing platform with the NovaSeq 6000 S4 Reagent kit V1.5. Bacterial genome assembly was performed using the SPAdes software (version 3.11) [34].

### 4.5. Antibiotic Resistance Genes and Phylogenetic Analysis

The ARGs for *Salmonella* strains were analyzed using the Center for Genomic Epidemiology (ResFinder tools). The relationship with non-typhoidal *Salmonella* isolates was evaluated using core-genome alignments and phylogenetic trees were constructed using Parsnp (neighbor-joining method)and visualized using the online tool (iTOL 6.5.7) [35].

## Figures and Tables

**Table 1:** Number of strains with resistance phenotypes.

Antibiotic Agent	2019 (*n* = 79)	2020 (*n* = 28)
Penicillin: Ampicillin	56 (70.9%)	20 (71.4%)
β-lactams combination: Amoxicillin/Clavulanic	3 (3.9%)	2 (7.1%)
Aminoglycosides: Gentamicin	36 (45.6%)	2 (7.1%)
Spectinomycin	23 (29.1%)	13 (46.4%)
Tetracyclines: Tetracycline	27 (34.2%)	21 (75.0%)
Florfenicol	59 (74.7%)	13 (46.4%)
Sulfafurazole	52 (65.8%)	16 (57.1%)
Trimethoprim/sulfamethoxazole	47 (59.5%)	13 (46.4%)
Ceftiofur	9 (11.4%)	-
Ceftazidime	2 (2.5%)	-
Enrofloxacin	43 (54.4%)	4 (14.3%)
Ofloxacin	17 (21.5%)	-
Meropenem	2 (2.5%)	-
Apramycin	-	-
Polymyxins: Colistin	5 (6.3%)	-
Mequindox	-	

**Table 2:** Number of *Salmonella* serovars.

Serotypes	In Total	Pig	Poultry
Potential monophasic variant of Typhimurium	34	27	7
Typhimurium	20	6	15
Derby	17	12	5
Rissen	13	9	4
Enteritidis	11	-	11
Apeyeme	3	-	3
Kentucky	2	-	2
Indiana	2	-	2
London	1	1	-
Meleagridis	1	-	1
Thompson	1	-	1
Reading	1	-	1
In total	107	55	52

**Table 3:** Antimicrobial resistance phenotype of 107 *Salmonella* isolates.

Antibiotic Agent	Abbreviation	Antibiotic Concentration Range (μg/mL)	Breakpoint Interpretive Criteria (μg/mL)			Results in Percentage (%)		
			S	I	R	S	I	R
Penicillin: Ampicillin	AMP	0–512	≤8	16	≥32	32 (29.9%)	0	75 (70%)
β-lactams combination: Amoxicillin/Clavulanic	AMC	0.5/0.25–256/128	≤8/4	16/8	≥32/16	76 (71%)	26 (24.3%)	5 (4.7%)
Aminoglycosides: Gentamicin	GEN	0.25–128	≤4	8	≥16	51 (47.7%)	19 (17.8%)	37 (34.6%)
Spectinomycin	STP	0–512	≤32	64	≥128	14 (13%)	26 (24%)	67 (62.6%)
Tetracyclines: Tetracycline	TET	0–512	≤4	8	≥16	17 (15.9%)	0	90 (84.1%)
Florfenicol	FFC	0–256	≤4	8	≥16	36 (33.6%)	0	71 (66.4%)
Sulfafurazole	SOX	0–512	≤256	-	≥512	39 (36.5%)	-	68 (63.6%)
Co-trimoxazole	SXT	0–32/608	≤2/38	-	≥4/76	47 (43.9%)	-	60 (56%)
Ceftiofur	EFT	0.12–256	≤2	4	≥8	96 (89.7%)	1 (0.9%)	10 (9.4%)
Ceftazidime	CAZ	0.12–256	≤4	8	≥16	97 (90.7%)	4 (3.7%)	6 (5.6%)
Enrofloxacin	ENR	0.01–32	≤0.25	0.5–1	≥2	18 (16.8%)	42 (39.3%)	47 (43.9%)
Ofloxacin	OFX	0.03–64	≤2	4	≥8	57 (53.3%)	33 (30.8%)	17 (15.9%)
Meropenem	MEM	0.03–10	≤1	2	≥4	104 (97.2%)	0	3 (2.8%)
Apramycin	APR	0–64	-	-	-	-	-	-
Polymyxins: Colistin	CL	0.12–256	≤2	-	≥4	95 (88.8%)	-	12 (11.2%)
Mequindox	NA	1–512	-	-	-	-	-	-

**Table 4:** Different resistance phenotypes between chickens and pigs.

Antimicrobial Agents	Pig (*n* = 55)	Chicken (*n* = 52)	*p*-Value
Ampicillin	40 (72.7%)	35 (67.3%)	0.6903
Amoxicillin/Clavulanic	2 (3.6%)	3 (5.8%)	0.6013
Gentamicin	17 (30.9%)	21 (40.4%)	0.3059
Spectinomycin	50 (90.9%)	18 (34.6%)	1.47
Tetracycline	54 (98.2%)	37 (71.2%)	<0.0005
Florfenicol	51 (92.7%)	21 (40.4%)	<0.0005
Sulfafurazole	50 (90.9%)	19 (36.5%)	<0.0005
Trimethoprim/sulfamethoxazole	46 (83.6%)	14 (26.9%)	<0.0005
Ceftiofur	1 (1.8%)	8 (15.4%)	0.0115
Ceftazidime	0 (0%)	2 (3.9%)	0.1420
Enrofloxacin	33 (60.0%)	14 (26.9%)	0.0005
Ofloxacin	7 (12.7%)	10 (19.2%)	0.3576
Meropenem	0 (0%)	2 (3.9%)	0.1420
Apramycin	-	-	
Colistin	0 (0%)	11 (21.2%)	0.0003
Mequindox	-	-	

**Table 5:** Different resistance phenotypes among S. 4,[5],12:i:- isolates.

Numbers	4,[5],12:i:- (*n* = 34)	
Antibiotic agent	Drug resistant	Multidrug resistant (*n* = 31)
Tetracyclines: Tetracycline	34 (100%)	31
β-lactam: Ampicillin	31 (91.2%)	31
Chloramphenicols: Florfenicol	31 (91.2%)	31
Aminocyclitols: Spectinomycin	31 (91.2%)	31
Sulfonamides: trimethoprim/sulfamethoxazole	30 (88.2%)	30
Fluoroquinolones: Enrofloxacin	26 (76.5%)	26
Sulfonamides: Sulfafurazole	24 (70.6%)	22
Aminoglycosides: Gentamicin	16 (47.1%)	16
Fluoroquinolones: Ofloxacin	7 (20.6%)	7
Cephalosporins: Ceftiofur	1 (2.9%)	1
Polymyxins: Colistin	1(2.9%)	1
β-lactams combination: Amoxicillin/Clavulanic	-	-
Carbapenems: Meropenem	-	-
Cephalosporins: Ceftazidime	-	-

## Data Availability

All genome assemblies of all strains were deposited in GenBank under the BioProject accession number PRJNA993584.

## Supplementary Materials

The following supporting information can be downloaded at: https://www.mdpi.com/article/10.3390/antibiotics12071178/s1, Table S1. MIC, serotypes and plasmid of 107 non-typhoidal *Salmonella* isolates.
